# Impact of the COVID-19 pandemic on mental health and family situation of clinically referred children and adolescents in Switzerland: results of a survey among mental health care professionals after 1 year of COVID-19

**DOI:** 10.1007/s00702-022-02512-6

**Published:** 2022-06-02

**Authors:** Anna Maria Werling, Susanne Walitza, Stephan Eliez, Renate Drechsler

**Affiliations:** 1 Department of Child and Adolescent Psychiatry and Psychotherapy, Centre for Child and Adolescent Psychiatric Research, Neuropsychology, University Hospital of Psychiatry Zurich, University of Zurich, Eisengasse 16, 8008 Zurich, Switzerland; 2 Swiss Society for Child and Adolescent Psychiatry and Psychotherapy, Bern, Switzerland; 3 Department of Psychiatry, University of Geneva School of Medicine, Geneva, Switzerland

**Keywords:** COVID-19, Children, Adolescents, Mental disorders, Clinical referral, Psychiatry

## Abstract

The aim of this study was to assess the impact of the COVID-19 pandemic on mental well-being of clinically referred children and adolescents and on their families from the perspective of mental health care professionals in Switzerland during the first year of the pandemic. Psychiatrists and psychologists for children and adolescents participated in an anonymous survey conducted online in April/May 2021. The survey was completed by 454 mental health care professionals, most of them working in outpatient clinics for child and adolescent psychiatry or in independent practices. Most participants indicated an important increase of referrals for depression (86.8% of respondents), anxiety disorders (81.5%), crisis interventions (76.2%), psychosomatic disorders (66.1%), suicidality (63.8%), and behavioral addictions, e.g., excessive gaming (64.6%). In contrast, referrals or treatment demands for disorders such as autism spectrum disorder or psychosis showed no substantial change or a slight decrease, respectively. According to 69% of respondents, patients experienced the highest psychological burden in January/February/March 2021. Family problems very frequently reported by mental health professionals were parents’ worries about loneliness/isolation of the child (49%), child’s education and academic future (33%), increased media use due to missing options of recreational activities (37.6%), as well as multiple stresses of mothers (36.3%). To conclude, the pandemic has substantially changed the pattern of disorders and the number of clinical referrals of children and adolescents with mental health problems, which has serious consequences for the treatment supply in Switzerland.

**Supplementary Information:**

The online version contains supplementary material available at 10.1007/s00702-022-02512-6.

## Introduction

Children and adolescents from all over the world have suffered from pandemic control measures and their consequences, such as school closures, social distancing, curfews, lack of leisure or group activities, limited possibilities for physical exercise, enhanced family conflicts due to home confinement, or financial insecurities. Population studies worldwide have shown that the pandemic had already dramatic effects on children’s and adolescents’ mental health, with increased depressive and anxious symptoms and increased suicidal ideation (reviews by Deolmi and Pisani 2020; Samji et al. 2021; Singh et al. 2020) and self-harm (Zetterqvist et al. 2021), psychosomatic complaints, worsened quality of life (Ravens-Sieberer et al. 2021), post-traumatic stress disorder (Marques de Miranda et al. 2020; Selçuk et al. 2021), eating disorders and weight gain (Juli et al. 2021; Woolford et al. 2021), as well as attentional problems (Mohler-Kuo et al. 2021), loneliness or isolation (Al Omari et al. 2021; Cost et al. 2021). Parents also experienced heightened stressors and burdens during the COVID-19 situation (Clemens et al. 2021; El-Osta et al. 2021; Huebener et al. 2021), and some of them were at risk to experience distress or to suffer from own mental problems (Tseng et al. 2021). Mothers, and single mothers in particular, were confronted with new challenges, and had to manage work at home, supervision of homeschooling as well as household chores. They were thus at risk for overload, exhaustion and depression (Calvano et al. 2021; McLaren et al. 2020; Taylor et al. 2021). Spending more time in confined spaces also resulted in more frequent family conflicts and sometimes led to physical and sexual abuse or maltreatment (Rodriguez et al. 2021; Sinko et al. 2021).

An increased need for treatment of mental health related problems or disorders in children and adolescents since the pandemic has been reported by several studies. In a survey among university hospitals of child and adolescent psychiatry from 22 European countries, a majority of respondents indicated an increase of cases with suicidal crises (more than 80%), depression (more than over 60%), anxiety (more than 70%), eating disorders (more than 60%) in February/March 2021, while in summer 2020, only a minority had reported an increased demand for treatment (Revet et al. 2021). Mental health related visits to emergency departments have also considerably increased during the pandemic in children and adolescents according to reports from several countries, such as Switzerland (Berger et al. 2022), Canada (Chadi et al. 2021), Italy (Vicari and Pontillo 2021) and United States (Leeb et al. 2020). According to a systematic review on the impact of the pandemic on child and adolescent mental health based on 61 articles (Panchal et al. 2021), anxiety symptoms and depression were among the most frequently reported symptoms, followed by irritability and anger. Adolescent age, female sex, lack of daily routine and excessive exposure to pandemic-related information and media or COVID-19 health-related stress were revealed as risk factors for increased mental problems (e.g.,Bhatia 2020; Kazi and Mushtaq 2021; Mohler-Kuo et al. 2021; Qin et al. 2021; Wang et al. 2022; Wirkner et al. 2022). However, for patients with pre-existent mental health disorders, results were mixed: several studies, especially when conducted at the beginning of the pandemic and during the lockdown, reported a certain relief of stress and decreased symptoms in a minority of patients with social anxiety, school anxiety, learning disorders and ADHD (Bobo et al. 2020; Cost et al. 2021; Melegari et al. 2021). For ADHD, a relief of symptoms, especially during the lockdown, has been reported in 25–30% of cases (Lavenne-Collot et al. 2021; Werling et al. 2021a), but on the other hand, an exacerbation of pre-existing symptoms during COVID-19 school closures has been described for children with ADHD, who also seemed stronger affected by COVID-19 related stress than neurotypically developed peers (Giallonardo et al. 2021). In adolescents with pre-existing mental disorders, the recommendation to adhere to strict hygiene measures and social distancing seem to have triggered or intensified obsessive–compulsive symptoms (Khan et al. 2022). According to a recent German study comparing a clinical and a community sample of children and adolescents, approximately 60–70% of children, adolescents and parents indicate an increase in mental burden, in contrast to 12% indicating a relief (Döpfner et al. 2021). The question has been raised whether the high increase of cases with mental problems are truly caused by the pandemic itself or are only triggered by the coronavirus crisis, induced by a situation of permanent stress and uncertainty, in individuals with a pre-existent vulnerability and poor resilience. Few studies have tried to distinguish between mental symptoms caused by the pandemic, pre-existent symptoms exacerbated by the pandemic, and those unrelated to the pandemic. For a group of adult psychiatric patients, Rohde and colleagues proposed an approach for the classifications of patients according to mental illnesses related or unrelated to the effects of the pandemic based on patient records (Enevoldsen et al. 2022; Jefsen et al. 2021; Rohde et al. 2020). Among the symptoms related to the effects of COVID-19, they identified anxiety symptoms, OCD-symptoms, and, in a smaller number of patients, autism- and ADHD-related symptoms, self-harm and unspecific stress. Several studies have shown an important increase of digital media use in children and adolescents, such as gaming, surfing in the internet or communicating on social media (Kawabe et al. 2020; Masaeli and Farhadi 2021), especially in the lockdown phase of the pandemic (Werling et al. 2021a, b). In adolescents with pre-existing mental health problems, very high media use has been linked to depression or worsening of psychopathology. On the other hand, digital media use had also protective effects during the pandemic, as it was often the only means to socialize with friends.

The aim of the present study was to assess the impact of the COVID-19 pandemic on the mental health of clinically referred children and adolescents and on the situation of their families during the first year of pandemic from the perspective of mental health professionals for children and adolescents. Here, we focus on the mental health-related consequences of the pandemic for patients and their families, while consequences for treatment supply and the situation of mental health professionals have been presented elsewhere (Werling et al. 2022b). In the present study we investigated whether mental health professionals had observed:

– Disorder-specific changes in the frequency of referrals during the pandemic,
– Mental disorders apparently caused or triggered by the pandemic, including observed changes in patients’ digital media-related behavior during the pandemic,
– Mental health problems that seemed specific for the pandemic or had not been encountered before in this form or intensity,
– Subgroups of patients who had been particularly affected by the pandemic,
– Peaks in the severity of psychological burden experienced in the course of the first year of the pandemic.
– Finally, we assessed how frequently mental health professionals had been confronted with pandemic-related complaints and difficulties reported by parents.

## Method and procedure

An anonymous online survey was conducted from April 22nd to May 24th, 2021 among psychiatrists and psychologists specialized in the treatment of children and adolescents. They were contacted via email addresses, which were extracted from public directories of professional societies in Switzerland. The invitation email explained the background and goals and contained the link to the survey. Two weeks later, a reminder was sent out. A small group of health professionals with no published email address was contacted by letter, which included a QR code leading to the online survey. This survey for mental health professionals was developed as a complement and follow-up to surveys for patients and their parents on the impact of the pandemic conducted by the authors (Werling et al. 2021a, b; Werling et al. 2022a, b). The online survey was available in German and French, which are the two most frequently spoken languages in Switzerland (main languages for approximately 85% of inhabitants). With few exceptions, where multiple choices were possible, participants had to select a single answer from several options. If they were unwilling or unable to answer the question (e.g., because not applicable to their work context), they could skip the question or specify the answer as a free text comment. The free text comments were analyzed and classified according to content analytical principles.

### Pandemic protection measures in Switzerland

Besides a lockdown period over approximately 8 weeks in spring 2020, COVID-19 protection measures were less restrictive in Switzerland compared to many other European countries. Only during this lockdown period, schools were closed or offered online teaching. The first schools already started on-site teaching in May 2020. Primary schools and many secondary schools remained open during a second serious wave of infections with a peak in autumn 2020. Restriction measures varied in intensity from school to school and changed during the study period. Most recreational activities for children and adolescents, including contact sports or other group activities, were not allowed throughout 2020. Vaccinations for the population started in late December 2020.

## Results

### Participants

A total of 454 mental health care professionals participated in the anonymous survey. Approximately 1800 invitations were sent out, which corresponds to a response rate of 24%. Among the participants, 38.5% were psychiatrists and 54.4% psychologists specialized in the treatment of children and adolescents, 3.9% indicated other professions (e.g., other therapist, pediatrician) (Table 1). Most of them were from the German speaking part of Switzerland (83.2%), followed by the French speaking part (14.4%) and the Italian speaking part (0.4%) (the latter had responded to the French version of the survey) (Table 1). Based on the language distribution in Switzerland (main language in the population: German 62.1%, French 22.8%, Italian 8%), the French speaking part was slightly underrepresented. Most of the participants (54.4%) worked in independent practice and 29.8% in a clinic for child and adolescent psychiatry and psychotherapy, mainly in outpatient clinics or specialized units (Table 1). Only *N* = 20 participants indicated to work in inpatient facilities. The majority of professionals (65.0%) indicated to treat the entire age range of children and adolescents. Approximately 13.7% indicated to treat primarily adolescents (13 years and older) and 12.4% school-age children (7–18 years). Only a few therapists indicated to treat primarily children under 7 years of age (1.8%), younger school-age children (7–13 years) (1.3%), or families (3.6%).

**Table 1:** Participants

	*N*	%
Professional group		
Child and adolescent psychiatrists	175	38.5
Child and adolescent psychologists	247	54.4
Other	18	3.9
Not stated	14	3.1
Work context		
Independent practice (for child and adolescent psychiatry or psychology)	247	54.4
Clinic for child and adolescent psychiatry	135	29.8
Outpatient services/day clinic	*115*	
Inpatient clinic/ward	*20*	
Other/not stated	72	15.8
Total participants	454	100

### The pandemic’s impact on referral, frequency, severity and as trigger of mental disorders

Participants were asked to indicate whether the occurrence of certain disorders or causes for referral had disproportionally increased or decreased during the pandemic. Marked differences between problem areas emerged: depression (46.7%), anxiety disorders (42.2%), as well as emergencies and acute crises (37%) showed strong increases according to the answers of participants, while only a very small number of participants indicated an increased occurrence of disorders, such as psychosis (Table 2). Among the neurodevelopmental disorders, learning disabilities seemed to occur currently more often than before the pandemic (slight increase indicated by 36.3% and strong increase by 19.8%), followed by aggressiveness/behavioral disorders (slight increase 36.3%, strong increase 10.1%). Many participants reported in free text comments that school refusal and social withdrawal had increased (*N* = 29; Supplement Table S1). Others reported increased apathy/lack of perspective/hopelessness (*N* = 10) or increased psychological problems of parents (*N* = 10) (Supplement Table S1).

**Table 2:** Changed distribution of reasons for referral during the pandemic (percent of responses)

	Strong decrease	Slight decrease	Unchanged	Slight increase	Strong increase	Not applicable/not stated
Family conflicts	0.2	0.2	20.7	46.7	23.8	8.4
Physical assault, sexual abuse	0.0	0.4	42.3	20.0	2.6	34.6
Self-harm	0.0	0.9	27.1	38.5	18.1	15.4
Emergencies, crisis intervention	0.2	0.2	10.4	39.2	37.0	13.0
Suicidality	0.0	0.9	20.7	39.4	24.4	14.5
Depression	0.2	0.0	9.0	40.1	46.7	4.0
Anxiety disorders	0.2	0.4	12.6	39.0	42.5	5.3
Psychosomatic disorders	0.2	0.4	24.2	41.0	25.1	9.0
Adjustment disorders, trauma	0.2	0.7	39.4	33.0	9.9	16.7
ADHD	0.2	2.4	63.0	17.0	4.2	13.2
Aggressiveness, behavioral/conduct disorder	0.0	2.2	36.3	36.3	10.1	15.0
Autism spectrum disorders	0.2	2.2	62.4	7.6	2.6	25.0
Learning problems	0.2	2.4	27.5	36.3	19.8	13.7
OCD	0.4	0.4	33.5	35.7	9.0	20.9
Eating disorders	0.2	0.2	32.2	29.7	11.9	25.8
Behavioral addictions (e.g., gaming)	0.2	0.0	17.0	38.8	25.8	18.3
Substance-related addictions (e.g., alcohol)	0.2	0.2	32.8	26.7	8.6	31.5
Psychosis, schizophrenia	0.0	0.0	46.3	8.6	1.1	44.1
Borderline personality disorder	0.0	0.0	43.8	16.7	2.6	36.8
Personality disorders in general	0.0	0.0	38.1	12.6	1.3	48.0

High percentage of “not-applicable”-responses may reflect a low incidence of a disorder or that many clinicians usually do not offer treatment for this group of patients

**Fig. 1:**
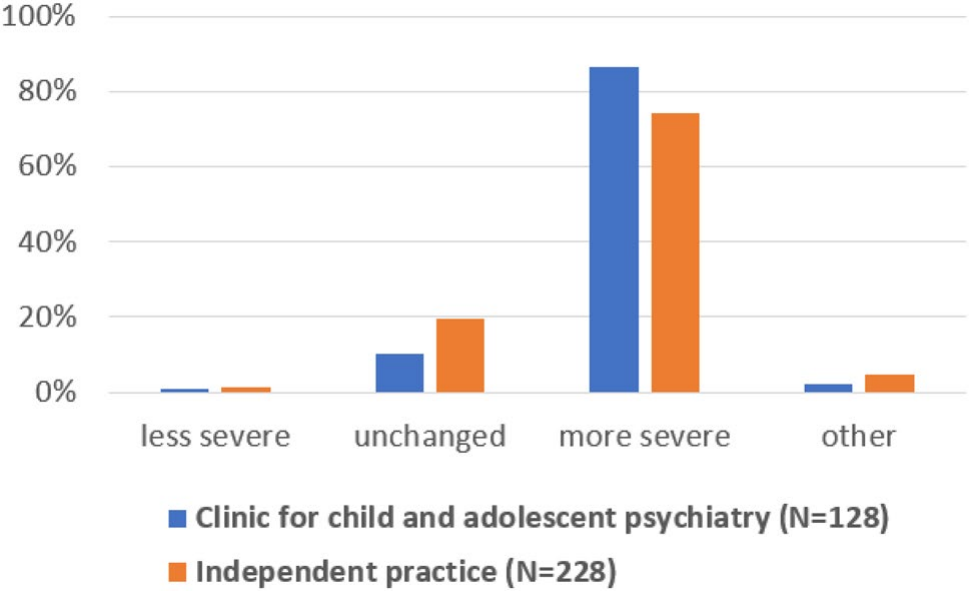
Changed severity of disorders as result of the pandemic as indicated by mental health professionals working in clinics or independent practices (frequency of responses in percent). Percentages refer to each professional group separately

When asked in another question whether according to their experience the severity of disorders had increased during the coronavirus crisis, most participants agreed that disorders and mental problems seemed to be slightly (55.7%) or much more severe (16.5%) than before the pandemic (Supplement Table S2). Some participants argued that problems may have seemed more severe, because waiting times for admission to treatment in psychiatric institutions had considerably increased and patients with severe and acute problems had to recur to independent practices instead or because resources within families were diminished (Supplement Table S2; free text comments). When estimation of severity was analyzed according to the work settings, 86% of professionals working in a clinic for child and adolescent psychiatry and 74% of professionals working in independent practice estimated that the severity of disorders had increased (somewhat more severe/much more severe) since the pandemic (Fig. 1). The difference in the frequency of responses between both groups was borderline significant (Chi^2^ = 7.764, *p* = 0.051) with professionals from clinics indicating more often an increase in severity.

In the next question, participants were asked to indicate, whether and which kind of disorders or mental problems had been caused/triggered by the pandemic in their opinion (Table 3). 67% percent of respondents indicated depression, followed by family conflicts (59.9%), psychosomatic disorders (39.9%), suicidality (37.7%), mutism/school refusal/social isolation (35.5%), addictive behaviors (23.8%), and eating disorders (19.2%) (Table 3). Among psychosomatic disorders, sleep disorders were mentioned most often (*N* = 154). Problematic gaming was the most frequently reported addictive behavior (*N* = 99), anorexia and bulimia (*N* = 70) were most frequently mentioned among eating disorders/problematic eating behaviors, and violence of a parent against the child (*N* = 49) was the most frequently indicated subcategory of domestic violence. Anxiety disorder (*N* = 17) and obsessive compulsive disorder (OCD) (*N* = 7) were additionally mentioned as triggered or caused by the pandemic in free text comments (Table S3). However, according to some other comments, the pandemic was also seen as catalyst rather than as cause of disorders, having a destabilizing effect on a previously effortful maintained balance (*N* = 11) (Supplement Table S3).

**Table 3:** Disorders or mental problems triggered/caused by the pandemic

	*N*	%
Depression	305	67.2
Family conflicts	272	59.9
Psychosomatic disorders^a)^	181	39.9
Sleep disorders	*154*	
Headache	*85*	
Other pain	*48*	
Suicidality	162	37.7
School refusal, social anxiety	161	35.5
Addiction, drug use^a)^	108	23.8
Alcohol	*46*	
Drugs (e. g. cannabis)	*66*	
Gaming (PUI)	*99*	
Eating disorders^a)^	87	19.2
Eating disorder in the strict sense (anorexia, bulimia)	*70*	
Uncontrolled food intake	*30*	
Severe weight gain	*30*	
Unfavorable eating habits (junk food)	*35*	
Loss of appetite	*22*	
Food refusal	*23*	
Domestic violence/abuse^a)^	63	13.9
Violence of the patient against the parent(s)	*33*	
Violence of a parent against the child	*49*	
Violence between parents	*40*	
Sexual abuse	*7*	
No, the pandemic was usually not a trigger	34	7.5
Other	67	14.7
Not applicable/not stated	32	7.1

^a)^If the category had been chosen, subcategories could be selected. Multiple answers were possible

### Problematic internet and digital media use

Approximately 20% of participants reported to have been confronted before the pandemic quite or very often with problematic use of the internet (PUI)/problematic gaming in their clinical work. During the pandemic, this number increased up to 41.2% (Table 4). When asked about changes in the media use of their patients during the pandemic, 36% of respondent had observed changes in digital media behavior up to a borderline problematic/addictive use quite or very often, and 16% indicated to have observed an important increase of digital media use above a pathological threshold quite or very often (Supplement Table S4).

**Table 4:** Frequency of “problematic internet use” (or “video game addiction” or “internet addiction”) as reason for referral or as suspected diagnosis before and since the pandemic

	Never	Seldom	Some-times	Quite often	Very often	Not applicable/not stated
	%	%	%	%	%	%
Before the pandemic	6.2	18.7	43.4	18.9	1.3	11.5
Since/during the pandemic	6.6	10.8	29.7	25.3	15.9	11.5

### Impact of the pandemic on pre-existent disorders, on patient subgroups and time course of psychological burden

When asked about the differential effects of the pandemic on pre-existing disorders or mental problems, the majority of participants indicated that effects were negative in general (55.7%), some reported that effects were individual, differing from case to case (23.6%), and only 1.3% reported that effects were positive rather than negative (Supplement Table S5). When asked which psychopathological disorder or group of patients had been particularly affected by the pandemic (Table 5), depression (67.4%) and anxiety disorders (63%) were indicated by far most often, followed by OCD (26%) and ADHD (25.8%).

**Table 5:** Patient groups/disorders that have been particularly affected by the pandemic

	*N*	%
Depression	306	67.4
Anxiety disorder	286	63.0
OCD	127	26.0
ADHD	117	25.8
Trauma, adjustment disorder	99	21.1
Eating disorder	86	18.9
Autism spectrum disorder	45	9.9
Psychosis	22	4.8
Other	14	3.1
Not stated	74	16.3

Multiple answers were possible

Conversely, when asked to indicate disorders or groups of patients that had been less affected or even had benefited during the pandemic, anxiety disorders (28%) and autism spectrum disorders (29.7%) were selected most often. General benefits were, however, often indicated as only short term (33%) and/or limited to the time period of the lockdown (30%) (Table 6). In free text comments, social anxiety was mentioned particularly often as temporarily improved in consequence to the pandemic (*N* = 12; Table 6).

**Table 6:** Have patient groups been less affected by the pandemic than others, or did they actually benefit?

	*N*	%
No one has benefited	30	6.6
Some patients have benefited		
Some patients with ADHD	64	14.1
Some patients with ASD	135	29.7
Some patients with anxiety disorder	127	28.0
Some patients with depression	19	4.2
Only during the lockdown (March/April 2020)	140	30.8
Only short term	150	33.0
Don’t know/not stated	64	14.1
Other	37	8.1
Free text comments (summary)		
School phobia/school absenteeism	5	
Social anxiety	12	
Learning disorders, school problems	3	
OCD	1	
Victim of bullying	2	
Other	12	

Multiple answers were possible

Regarding differential effects of the coronavirus crisis on age groups or gender, a majority (63.9%) found that adolescents had been most severely affected by the pandemic (Table 7). Only 10.1% of participants viewed children between 7 and 12 years as most affected. Most participants estimated that boys and girls had been equally severely affected (65.4%). Nevertheless, 14.1% of the participants found that girls had been more severely affected, while only 3.3% indicated boys as more affected (Supplement Table S6).

**Table 7:** Age group most affected by the pandemic

	*N*	%
Younger children (up to 7 years)	7	1.5
Primary school age (7–12 years)	47	10.1
Adolescents (13 years and older)	290	63.9
All about the same	51	11.2
Don’t know/not stated	56	12.2

**Fig. 2:**
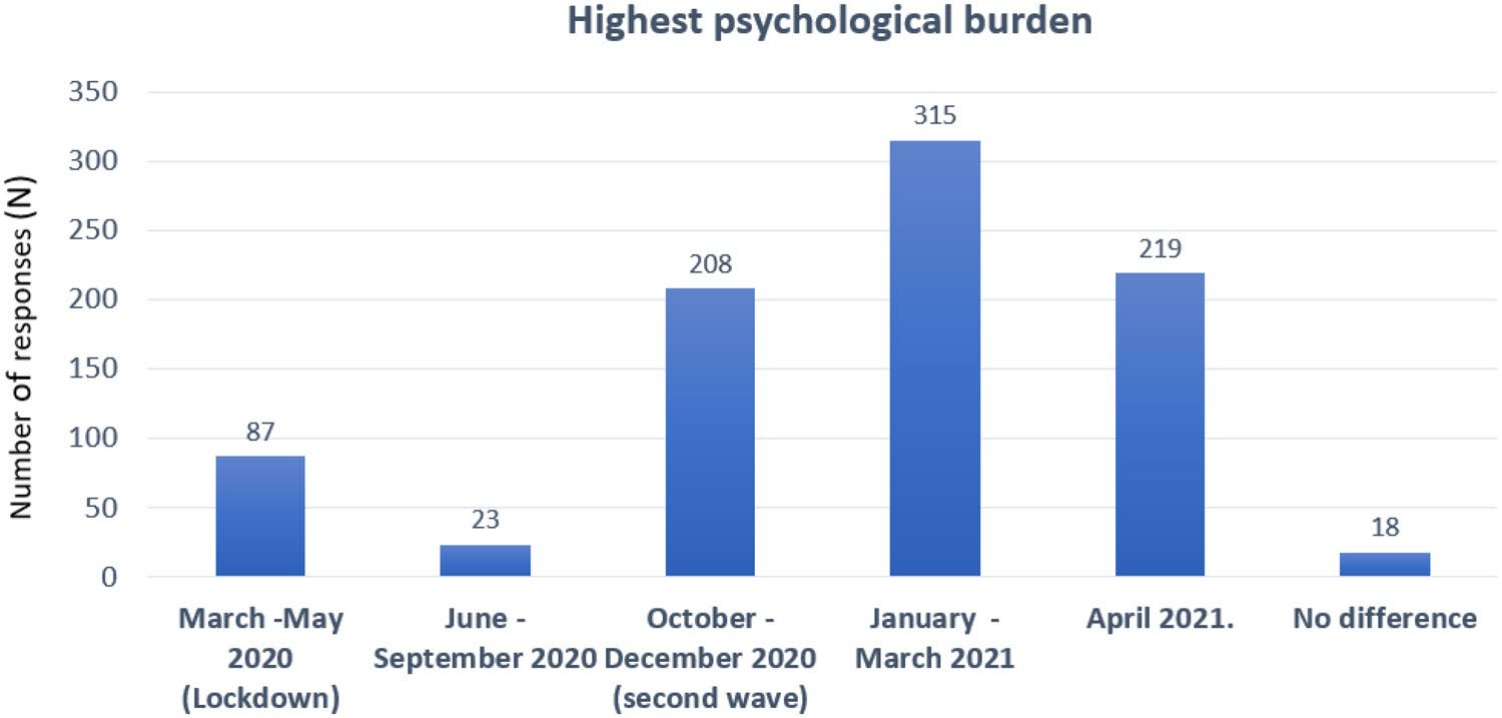
Time period of the pandemic with particularly high psychological burden for patients (frequency of responses). Multiple responses were possible

Participants were also asked to indicate the time period in the course of the first year of pandemic, which had been psychologically the most difficult for the patients. Winter 2021 (January, February, March 2021) emerged as the most difficult time period, as indicated by 69% (*N* = 315) of respondents, followed by autumn 2020 (54.2%; *N* = 208). The actual lockdown period (March/April 2020) was rarely seen as particularly stressful (19.2%, *N* = 87) (Fig. 2, Supplement Table S7).

### Pandemic-specific problems

Participants were asked to indicate whether certain problems or disorders encountered during the pandemic were new or different compared to pre-COVID-19 times. 42% of participants responded affirmatively and described pandemic-specific problems in free text comments (Table 8). In the analysis of these comments, three pandemic-specific topics emerged: 1. future-related anxiety/lack of perspective and concrete difficulties in the transition from school to apprenticeship/further education/profession (*N* = 49), 2. school absenteeism, school anxiety and avoiding returning to school (*N* = 25), and 3. loneliness and isolation (*N* = 24). Other relatively common problem areas were the constraints and fears related to hygiene rules and fear of infection (*N* = 18), and increased consumption of digital media (*N* = 16) (Table 8).

**Table 8:** Pandemic-specific problems/changes (*N* = 193) (free text summary)

	*N*
Fear of the future, education more difficult, career entry more difficult, lack of perspective, disorientation, “stolen youth”	49
School absenteeism, school anxiety, fear of returning to school	25
Lack of social contact, loneliness	24
Hypochondria/anxieties/compulsions intensified or newly developed because of pandemic hygiene rules and attention to symptoms	18
Digital media consumption, digital media addiction	16
Obsessive–compulsive patients had more time to pursue compulsions, anxiety and compulsions increased or new, panic attacks	15
Suicidality/depression	13
Lack of sports opportunities exacerbate hyperactivity and weight problems, no recreational opportunities	11
Family conflicts, domestic violence	11
Lack of school and parental guidance, lack of structure	10
Financial problems, parents’ loss of work promotes conflicts	7
Loss of stabilizing factors (e.g., meeting friends, future plans, meeting grandparents) leads to decompensation	6
Difficulty in detaching from parents; adolescents feeling overly controlled by parents	5
Fear that family members might die or be infected, topic of death, mourning	5
Activity buildup impossible for patients with depression	4
Positive aspects: Families benefit from quiet time at home, relief from social pressure for autistic patients, fathers are more present	4
Drugs	4
Insufficient regulatory control of families at risk	1
Long-COVID syndrome	1

### The situation of parents during the pandemic

When participants were asked about problems expressed by parents during the pandemic more frequently than before, loneliness/isolation of the child (49%) and worries about the child’s education and academic future (33%) emerged as most frequent concerns expressed by parents. Complaints about general isolation and loneliness of the family, financial problems and problems related to the lack of child care options were also expressed by parents more often than before the pandemic (Table 9).

**Table 9:** Frequency of family problems reported by parents before and since the pandemic

	Much less	A little less	No change	A little more	Much more	Not stated
	%	%	%	%	%	%
Conflicts between patient and parent(s)	0.2	0.7	13.9	56.6	19.2	9.5
Conflicts between parents	0.0	0.4	17.2	53.1	15.0	14.3
Domestic violence	0.0	0.4	23.6	36.1	3.5	36.3
Financial problems/job loss	0.0	0.0	15.2	48.7	19.4	16.7
General isolation of the family	0.0	0.2	11.5	48.0	24.7	15.6
Isolation/loneliness of the child/adolescent	0.0	0.2	3.7	39.4	49.1	7.5
Lack of child care options	0.7	0.2	18.7	36.3	25.6	18.5
Concern about child's future/education	0.0	0.9	15.4	38.8	33.0	11.9
Mental problems of one parent	0.0	0.2	34.1	34.8	7.5	23.3
Addiction/alcohol problems of one parent	0.0	0.0	37.0	25.3	3.3	34.4

When asked about problems reported by parents which are directly related to the pandemic and its consequences, mothers’ multiple workloads regarding office work at home, household activities, and caregiving responsibilities (36.3%), children’s increased media time due to missing alternative leisure activities (37.4%), and parents being overwhelmed in taking care of homeschooling (32.4%) were mentioned very often (Table 10). In free text comments, the loss of family contacts and lacking support by grandparents were also mentioned as frequently encountered problems, among others (*N* = 11) (Supplement Table S8).

**Table 10:** How often did parents report the following effects of the pandemic?

	Never	Rarely	Some-times	Often	Very/extremely often	Not stated
	%	%	%	%	%	%
The child is left alone at home (lack of child care possibilities)	6.8	18.5	34.6	15.4	10.4	14.3
Parents cannot adequately take care of the child due to work at home	4.4	11.7	33.0	23.3	13.3	14.3
Mothers suffer from the multiple burdens (child care, home office, household)	0.7	2.9	16.7	31.9	36.3	11.5
Fathers suffer from the multiple burdens (child care, home office, household)	2.2	12.8	31.9	25.1	13.7	14.3
Parents feel overwhelmed by supervision of homeschooling	0.0	3.1	21.1	32.8	32.4	10.6
Parent–child conflicts because of homeschooling	0.7	3.7	22.9	30.4	29.3	13.0
Parents allow excessive media use to keep child occupied	0.0	3.1	17.6	29.3	37.4	12.6
Learning problems due to homeschooling	0.4	5.5	20.0	31.3	30.0	13.0

## Discussion

In the present survey, we investigated the impact of the first year of the COVID-19 pandemic on mental health and family problems of clinically referred children and adolescents from the perspective of mental health care professionals in Switzerland.

### Disorder-specific changes in the frequency of referrals during the pandemic

This survey revealed an important change in the disorder-specific pattern of referrals. A strong increase was indicated for depression and anxiety disorders by most respondents (over 40%), followed by crisis intervention, suicidality, psychosomatic complaints and behavioral addictions. This is well in line with other studies reporting an increase in the aforementioned disorders in children and adolescents during the pandemic (Panchal et al. 2021). On the other hand, the majority of respondents reported no relevant change in the number of referrals for autism spectrum disorders or only very small increases for ADHD or psychosis. A decrease of referrals was not reported for any of the disorders. One may assume, in consequence, that the pandemic had a selective impact on certain disorders, especially those associated with internalizing symptoms, but not on psychopathology in general. It has been reported that for some children with ADHD or ASD, containment measures such as staying at home, reduction of social contacts and having no longer to conform to school discipline were experienced as a reduction of pressure and social stress which led to stress reduction and consequently to a relief of symptoms (Bruining et al. 2020; Bobo et al. 2020; Cost et al. 2021). However, this is not reflected by the number of referrals which did not decrease during the pandemic. Among the neurodevelopmental disorders, a relevant increase in referrals was reported for learning disorders and, to a smaller extent, behavioral disorders/aggressiveness. The former may be explained by the fact that children with learning problems might have been unable to keep up with their studies during remote learning or that deficits may have become more visible for parents who had to supervise their child’s schoolwork during homeschooling measures. Increased behavioral dysregulation, anger and irritability during the pandemic have been described in children and adolescents during the pandemic (Mohler-Kuo et al. 2021; see Panchal et al. 2021), often in interaction with enhanced parental stress (Andrés et al. 2022; Montirosso et al. 2021). Referrals for OCD, eating disorders, behavioral addictions and substance had also increased, in agreement with studies on pandemic effects on eating disorders (Otto et al. 2021; Schwartz and Costello 2021), OCD (Alhujaili et al. 2021; Sowmya et al. 2021), substance use disorder (Sen et al. 2021), problematic digital media use (Masaeli and Farhadi 2021). The number of participants who indicate frequent referrals for problematic use of the internet/problematic gaming has more than doubled during the pandemic.

Interestingly, for disorders such as psychosis or schizophrenia, most health professionals indicated no change in the number of referrals. This is consistent with literature according to which patients with internalizing/affective disorders are more affected by the pandemic than patients with psychotic disorders (Gul and Demirci 2021; van Loon et al. 2021). The latter have shown, however, a higher risk of being infected with COVID-19 (Karaoulanis and Christodoulou 2021). The high number of missing responses in this category may be explained by its lower prevalence and by the fact that many child and adolescent psychotherapists do not treat patients with psychosis. When only valid responses are considered, about 20% of remaining respondents reported a slight increase of psychoses during the pandemic. This may be indicative of a heightened psychological distress under COVID-19 conditions in this vulnerable group. An increase in prevalence of psychosis or (reactive) brief psychotic episodes during the pandemic has been described for adults (Segev et al. 2021). However, a relative increase was reported for all disorders and may also reflect a generally increased demand for treatment due to the distress during the corona crisis. All in all, a pattern emerged, with an important increase of referrals for internalizing disorders including depression, anxiety disorders and psychosomatic problems, and also for domestic conflicts, crisis intervention, suicidality, and behavioral addictions, whereas referrals of disorders, such as psychosis and ASD remained relatively stable.

### Mental problems caused or triggered by the pandemic

When we asked for problems caused or triggered by the pandemic, depression and family conflicts were explicitly attributed to the pandemic by the majority of participants. Psychosomatic disorders, especially sleep problems, suicidality, (social) anxiety, were still linked to the pandemic by more than a third of participants. Addictive behaviors and eating disorders were considered to be caused or triggered by the pandemic by approximately 20%. These percentages may partly be confounded with differences in the prevalence of the disorders or by the fact that certain disorders are usually treated by a small number of specialists. General anxiety disorder was not included among the response options but was mentioned by several respondents in free text comments as caused by the pandemic. In contrast, the number of mental health professionals who explicitly denied a relation between the pandemic and the increase of mental problems was very small (7.5%). Nevertheless, some participants assumed in free text comments a pre-existent vulnerability in patients that may have prevented a successful coping with the corona crisis and which led to the need for treatment.

### Severity of disorders

There was a general agreement among participants that the severity of disorders had increased during the pandemic, which may be related to the reported high occurrence of acute crises and suicidality and which is in accordance with studies showing an exacerbation of symptoms during the pandemic (Tanir et al. 2020). Participants working in clinics for child and adolescent psychiatry reported a slightly higher increase in severity than those working in independent practice. This probably reflects the fact that usually the more severe, complex, or acute cases are seen and subsequently treated in clinics for child and adolescent psychiatry rather than in independent practice settings. As emergencies dramatically increased during the corona crisis in Switzerland among children and adolescents with mental health problems (Berger et al. 2022; Werling et al. 2022b), along with a shortage of treatment places and longer waiting time before admission to treatment, many mental health professionals working in independent practice must have been confronted with severe cases, which in normal times would rather have been treated in clinics for child and adolescent psychiatry. This was also reported in the free text sections.

The question arises whether the increased rate of affective disorders, acute crises and suicidality reported by professionals in this survey is reflected by an increase of emergency consultations and suicide rates as more objective measures. According to a recently published Swiss retrospective cohort study of a child and adolescent psychiatric out-patient emergency facility based on electronic patient records, the demand of emergency service considerably increased during the pandemic (Berger et al. 2022). Compared to before the pandemic, emergency bridging interventions increased by 230% and inpatient admissions of minors to adult psychiatric inpatient units, necessary because of lacking treatment capacity in child and adolescent psychiatry, more than doubled, due to significant increase of suicidality and self-harm behavior. Other studies also reported an increase of minors presenting with suicidality in emergency departments (Carison et al. 2022) or reported higher rates of suicide attempts among adolescents psychiatrically hospitalized during the pandemic in comparison to the year before (Thompson et al. 2021). Stressors during the pandemic-like loneliness, social isolation or financial burden amongst others were made responsible for the increased suicide risk (e.g., Sher 2020).

### Pandemic-specific effects

Specific topics that emerged as new and direct effects of the pandemic, were related to consequences of confinement: the fear of the future/lack of perspective reported by adolescents, school anxiety and absenteeism, and lack of social contact, loneliness. Other, less frequently reported topics were newly developed or intensified compulsions because of pandemic hygiene rules and increased self-observation for possible signs of infection.

### Differential effects on subgroups of patients and of time periods

The majority of professionals assumed that the pandemic had hit adolescents particularly hard, which has been confirmed by other (epidemiological) studies (Hawes et al. 2021). In adolescence, social relationships with peers outside the core family become a high priority. Consequently, since pandemic’s restriction measures focusing on social distancing, it is not surprising that adolescents seem to have suffered most during this time. In terms of gender effects, the majority (64%) of professionals claimed that girls and boys had been affected to the same extent by effects of the pandemic, and only 14% of the participants indicated that girls had been more severely affected than boys (while the opposite was indicated by 3%). This is a relatively small difference compared to other results showing that negative effects of the pandemic on mental health are more severe in females (Halldorsdottir et al. 2021; Liu et al. 2022; Ma et al. 2021). However, in the present sample, participants refer to patients who all suffered from mental health problems, irrespective of their gender. Thus, in a clinical sample, an advantage for boys in adapting to the pandemic may still exist, but to a lesser degree than in epidemiological samples. January/February/March 2021 emerged as the time period with the highest psychological burden for patients, which was slightly delayed to the peak of the “second wave” of COVID-19 infections in October/November 2020 in Switzerland. The lockdown period in March/April 2020 and the summer months of 2020 were not associated with a particular high burden. This time course is in agreement with reports from Germany (Döpfner et al. 2021). When asked to indicate the subgroups of patients/disorders that had been most affected by the pandemic, depression and anxiety disorders were indicated by most participants. In a German study, patients with depressive disorders, especially girls, were also identified as the group with the highest psychological burden (Gilsbach et al. 2021). ADHD and OCD were still indicated by approximately 25% as among the most severely affected. On the other hand, ADHD was also among the disorders that were supposed to have benefited but this was only indicated by a small number of participants (14%), compared to ASD (29%) or anxiety disorders (28%). This confirms the inconsistent effects of the pandemic on ADHD and anxiety disorder reported previously, with improved symptoms due to a relief of pressure during school closures and limited social contacts in some patients, but increased symptoms under pandemic conditions in others.

### Pandemic-related complaints and difficulties reported by parents

Isolation/loneliness of the child and concern about the child’s education/academic and professional future emerged as the worries reported most often by parents since the pandemic, followed by domestic conflicts, the isolation as a family and lack of child care options due to confinement measures. Other frequently reported effects on families were that parents felt constrained to allow excessive media use in the absence of other leisure activities, multiple burdens imposed by pandemic restrictions especially on mothers, and parents feeling overwhelmed by the supervision of homeschooling tasks. The burden experienced by mothers under COVID-19 has been reported in the literature (Calvano et al. 2021; McLaren et al. 2020; Taylor et al. 2021), and mothers of children with depression or developmental disorders are known to be under extra strain (Babore et al. 2021; Burnett et al. 2021; Chafouleas and Iovino 2021; Wang et al. 2021).

### Support and measures taken to date during the pandemic

Since the pandemic with its restrictions has also led to a strain on mental health and psyche in the general population, several support services had been already expanded during the pandemic in Switzerland. Various low-threshold psychosocial counselling services, information platforms and websites were implemented for the prevention of psychiatric disorders and maintenance of mental health for the general population of all ages (ECDC 2020). For the protection of the mental health of the Swiss population, providers such as “Dargebotene Hand”, “Pro Juventute” and “Pro Mente Sana” had expanded their counselling services since spring 2020 (Stocker et al. 2021). The Federal Office of Public Health (FOPH) additionally supported two information platforms for the general population (www.dureschnufe.ch and www.reden-kann-retten.ch).

For patients, especially for children and adolescents with pre-existing psychiatric disorders, the pandemic was particularly challenging. However, medical support in general and therapeutic care were limited to (acute) crises or carried out partly via telemedicine. To continue to provide necessary care, minor patients were triaged to adult psychiatry for inpatient treatment even more frequently than before the pandemic. In addition, special temporary regulations had been introduced for the billing of telemedicine consultation.

Taken together, the effects of the pandemic reported by parents and the new and pandemic-specific topics that emerged in therapy, such as loneliness, fear of the future, worry about educational goals, isolation of the family, suggested a direct effect of the confinement measures on the deterioration of mental health. It may be difficult to distinguish whether the apparent rise of mental illness during the corona crisis is entirely caused by the pandemic, triggered by the pandemic in individuals with heightened pre-existent vulnerability, or simply an exacerbation of pre-existent symptoms. In this study, most mental health professionals perceived anxiety disorder, depression, and suicidal crises in patients treated during the first year of pandemic as triggered or caused by the corona crisis. Also, adolescence is in general a period of heightened vulnerability and emotional imbalance (Larsen and Luna 2018), which may more easily lead to reactive mental problems than at other periods of life, and which challenges a clear differentiation between typical and psychopathological reactions.

### Limitations

The first limitation is that all information is based on subjective reports and not on objective patient statistics provided by the individual therapists. In addition, data relating to pre-COVID condition were collected retrospectively and may thus be subject to bias. Another limitation is that we did not include anxiety disorders among the preselected categories in the question on mental disorders triggered by the pandemic. Furthermore, the response rate of 24% was rather low, although still remarkable for a group of people with extreme work overload during pandemic times. In consequence, the representativity of the study is uncertain. Finally, a possible impact of the mediatization of the pandemic and de-stigmatization of psychiatric and psychological care on the increase of referrals cannot be ruled out. Also, opinions of professionals may have been influenced in the same way.

## Conclusions

In this study, based on reports by mental health professionals, it was shown that during the first year of pandemic, the number of clinically referred children and adolescents to psychiatric or psychological treatment had substantially increased and the pattern of disorders had changed. In particular internalizing disorders such as depression, anxiety disorders, acute crises including suicidality and psychosomatic problems along with problematic internet use were treated more frequently than before the pandemic, while the frequency of other disorders, such as psychosis or autism spectrum disorder, remained relatively stable. For most mental health professionals, the reported changes seemed directly related to the effects of the corona crisis and its restrictions. As the development of the COVID-19 pandemic or other crises is not predictable, measures should be taken to prevent similar catastrophic effects on youth in the future. This includes measures against the shortage of treatment supply in mental health care for children and adolescents, as well as measures to avoid social isolation of children and adolescents, to provide better support for families during crises, and to identify adolescents at risk early.

## Supplementary Information

Below is the link to the electronic supplementary material.

Supplementary file1 (DOCX 30 KB)
