# Supplementary material for: Impact of the COVID-19 pandemic on mental health and family situation of clinically referred children and adolescents in Switzerland: results of a survey among mental health care professionals after 1 year of COVID-19
Source: J Neural Transm (Vienna). 2022 Jun 2;129(5-6):675–88. doi: 10.1007/s00702-022-02512-6 (PMC9160518; doi:10.1007/s00702-022-02512-6)
Supplement: Supplementary file 1 — Supplementary file1 (DOCX 30 KB) [file 702_2022_2512_MOESM1_ESM.docx]

**Supplement**

**Impact of the COVID-19 pandemic on mental health and family situation of clinically referred children and adolescents in Switzerland: Results of a survey among mental health care professionals after one year of COVID-19**

Anna Maria Werling, Susanne Walitza, Stephan Eliez, Renate Drechsler

| **Table S1. Changed distribution of reasons for referral during the pandemic (summary of free text comments)**   \|  \| N \| \| --- \| --- \| \| School refusal, social withdrawal \| 29 \| \| Depression/ anxiety \| 12 \| \| Problems of the parents/ psychological illness/ burdening of the parents \| 10 \| \| Apathy/ resetting own needs/ exhaustion symptoms/ hopelessness/ lack of perspective \| 10 \| \| Loneliness/ loss of contacts \| 7 \| \| Family conflicts/ problems (also domestic violence) \| 6 \| \| Relapses/ deterioration of stabilized patients \| 5 \| \| Increased consumption (drugs, media, pain control medication) \| 4 \| \| Disconnected at school \| 3 \| \| Aggressiveness \| 3 \| \| Suicidal tendencies, self-harm \| 2 \| \| Dissociative states \| 2 \| \| Negative influence of harmful internet forums \| 1 \| \| Increased stress in pregnant women \| 1 \| \| Concentration problems \| 1 \| \| Long-COVID in adolescents \| 1 \| |
| --- | --- | --- | --- | --- | --- | --- | --- | --- | --- | --- | --- | --- | --- | --- | --- | --- | --- | --- | --- | --- | --- | --- | --- | --- | --- | --- | --- | --- | --- | --- | --- | --- | --- | --- |
|  |

**Table S2. Changed severity of disorders/ mental problems during the pandemic as indicated by mental health professionals**

|  | N | % |  |  |
| --- | --- | --- | --- | --- |
| much less severe | 1 | 0.2 |  |  |
| slightly less severe | 3 | 0.7 |  |  |
| unchanged | 70 | 15.4 |  |  |
| slightly more severe | 253 | 55.7 |  |  |
| much more severe | 75 | 16.5 |  |  |
| Other/ not stated | 52 | 11.5 |  |  |
| ***Free text comments (summary)*** | | | | N |
| Anxiety and OCD more severe, with concomitant relief from school closure | | | | 9 |
| Crises more complex, due to fewer resources within the family | | | | 8 |
| Decreased coping strategies of patients under the pandemic, also due to lack of other occupations | | | | 6 |
| Shift of severe cases from clinics of psychiatry to independent practices, because of missing treatment places in psychiatry | | | | 4 |
| Varies from patient to patient, depending on disorder | | | | 4 |
| Eating disorders more severe | | | | 3 |
| Self-harm/suicidality/depression more severe | | | | 2 |
| Crises in personality disorders | | | | 2 |
| Depends on timing. More depression/suicidality in 2nd wave | | | | 2 |
| Enrollment profile changed: previously ADHD 40%; now affective disorders/ mixed disorders 80%. | | | | 1 |
| Pandemic acts like a burning glass | | | | 1 |

**Table S3. Disorders or mental problems caused/ triggered by the pandemic**

**(summary of free text comments)**

|  | N |
| --- | --- |
| Anxiety, anxiety disorders | 17 |
| Pandemic more as amplifier, catalyst, not as trigger | 11 |
| OCD (e.g., compulsive washing) | 7 |
| Problematic digital media use | 3 |
| Depression | 2 |
| Lack of drive | 1 |
| School absenteism | 1 |
| Learning disorder | 1 |
| Other | 12 |

**Table S4. Frequency and nature of changes in the use of digital media during the pandemic as observed in patients by mental health professionals ^1^**

|  | Never | Seldom | Some-times | Quite often | Very often | Not stated |
| --- | --- | --- | --- | --- | --- | --- |
|  | % | % | % | % | % | % |
| Decrease | 50.0 | 22.7 | 3.3 | 0.7 | 0.0 | 23.3 |
| Increase, but no problematic use | 0.9 | 5.3 | 34.8 | 34.8 | 10.1 | 17.9 |
| Significant increase, borderline problematic/ addictive use | 3.3 | 11.0 | 33.9 | 31.1 | 5.3 | 15.4 |
| Significant increase with addictive character | 10.4 | 21,.1 | 30.6 | 14,.1 | 2.6 | 21.1 |

^1^Refers to digital media use as leisure activity

**Table S5. How has the pandemic affected pre-existing disorders/ symptoms?**

|  | N | % |
| --- | --- | --- |
| No/ very small effects | 22 | 4.8 |
| Rather positive effects | 6 | 1.3 |
| Rather negative effects | 253 | 55.7 |
| Rather no effect on symptoms, but new, different problems | 29 | 6.4 |
| Variable effects, no general trend | 107 | 23.6 |
| Not stated | 37 | 8.1 |

**Table S6. Which gender has been particularly affected by the pandemic?**

|  | N | % |
| --- | --- | --- |
| Girls | 64 | 14.1 |
| Boys | 15 | 3.3 |
| Same for all | 297 | 65.4 |
| Other/not stated | 81 | 18.8 |

**Table S7. Time period of the pandemic with particularly high psychological burden for patients**

|  | N | | % |
| --- | --- | --- | --- |
| March/ April/ May 2020 (lockdown) | | 87 | 19.2 |
| June/ July/ August/ September 2020 | | 23 | 5.1 |
| October/ November/ December 2020 (“second wave”) | | 208 | 54.2 |
| January/ February/ March 2021 | | 315 | 69.4 |
| April 2021 | | 129 | 28.4 |
| No difference | | 18 | 4.0 |
| Don't know/ not stated | | 39 | 8.8 |

Multiple answers possible

**Table S8. Other family problems caused by the pandemic that are commonly reported by parents (summary of free text comments)**

|  | N |
| --- | --- |
| Lack of family contacts, care possibilities (grandparents) | 11 |
| Separation of parents, couple conflicts | 9 |
| Parental exhaustion, lack of perspective | 8 |
| Parents and children lack compensatory opportunities; no possibility to relieve stress | 8 |
| Family conflicts, lack of opportunities for young people to detach, violence | 5 |
| Boredom, lack of structure for the child | 5 |
| Fear for grandparents, illness and death in the family | 4 |
| Stress due to work at home, financial worries, spatial confinement | 4 |
| School problems/ homeschooling/ parents realize school problems | 3 |
| Overprotective behavior due to fear of infection | 1 |
| Overprotective behavior of parents due to low media skills | 1 |
| Other | 9 |

**Table S9. Overview of the questionnaire to the professionals**

1. Does the clientele differ who is seeking treatment since the pandemic than before? Are there proportionately more patients with certain disorders or problems than before the pandemic? Are there patients with certain disorders that you treat (or can treat) proportionately less than before the pandemic? - Please indicate whether the proportion of the following disorders has changed in your patients since the pandemic *(see Table 2: “Changed distribution of reasons for referral during the pandemic”)*
2. Have you observed disorders or problems that in your opinion have been triggered by the pandemic? *(see Table 3: Disorders or mental problems triggered/ caused by the pandemic)*
3. How many times was "problematic Internet use" (or "video game addiction" or "Internet addiction") stated as a reason for referral or a suspected diagnosis among your patients? *(see Table 4: Frequency of "problematic internet use" (or "video game addiction" or "internet addiction") as reason for referral or as suspected diagnosis before and since the pandemic.*
4. Are there any disorders which have been particularly affected by the pandemic? *(see Table 5: Patient groups/ disorders that have been particularly affected by the pandemic (percentages of response))*
5. In your experience, are there groups of patients who have been less affected by the pandemic or perhaps have benefitted?  *(see Table 6)*
6. According to your experience, which age group has suffered particularly from the pandemic? *(see Table 7: Age group most affected by the pandemic)*
7. Are there any other disorders or problems that since the pandemic occur more frequently in your patients than before?” (*see Table 8: Pandemic-specific problems / changes (N=193) (free text summary)*
8. Since the pandemic, which of the following stresses have been reported by parents more frequently or less frequently than before? (see Table 9: F*requency of family problems reported by parents before and since the pandemic)*
